# Reproducibility assessment of Dextrusion: A deep learning pipeline for detecting epithelial cell extrusion events

**DOI:** 10.1111/jmi.70101

**Published:** 2026-05-06

**Authors:** Nicholas D Condon, Jian Xiong Wang, Alessandro A Felder

**Affiliations:** ^1^ Institute for Molecular Bioscience The University of Queensland Brisbane Australia; ^2^ Neuroinformatics Unit, Sainsbury Wellcome Centre and Gatsby Computational Neuroscience Unit University College London London UK

**Keywords:** deep learning, image analysis, microscopy, repeatability, reproducibility

## Abstract

Reproducibility is essential for scientific software but can be hindered by technical and maintenance challenges. As part of the Global Bioimage Analyst Society reproducibility initiative, we evaluated Dextrusion, a deep learning pipeline for detecting epithelial cell extrusion events published by Villars et al. The code is openly available and supported by example datasets, ImageJ macros, and Jupyter notebooks. We can independently confirm the repeatability (using the provided example data) and reproducibility (using new semi‐synthetic confocal data) to detect cell death and division events using Dextrusion. This mini‐review highlights both the strengths of Dextrusion, including transparency and performance, and the wider challenges of long‐term sustainability and platform compatibility in publishing scientific software.

## 1 INTRODUCTION

Reproducibility is essential to science. Software tools used to publish scientific findings must help ensure reproducibility. This requires high‐quality documentation so the wider research community can use the software effectively. Ensuring the tool works (especially if it depends on other libraries and packages) in the hands of many researchers with varying skills, experiences and operating systems is especially challenging. It is also not clear how long the tool is expected to work and be maintained for, as key dependencies might be updated, changed or deprecated, leading to bugs, or broken elements of the tool. Scientific publications are static; however, many publishers rightly expect data and code to be shared alongside a publication. Code is not static, and requires ongoing maintenance and upkeep to remain usable – ironically, updating the software or its dependencies may render original results non‐reproducible, unless a containerised version with the original version is provided. Who is responsible for this, who funds this work, and who checks or reviews this is not clear or established.

As part of the inaugural Global Bioimage Analyst Society (GloBIAS) meeting held in Gothenburg, Sweden in 2024, attendees formed small groups to test the repeatability and reproducibility of several recently published manuscripts that contained a significant image analysis pipeline or software tool. This article will review the 2023 Dextrusion publication by Villars et al. in Development, presented as a ‘Techniques and Resources’ submission type,^1^ against the backdrop of current software practice in the bioimage analysis community.

Dextrusion is a deep learning‐based pipeline for the automatic detection of particular cellular events, particularly focusing on cells being extruded from epithelial tissues. The tool utilises a recurrent neural network called DexNet that classifies image sequences detected from the input movie files into categories such as cell death or division (as well as sensory organ precursor cells). The detected events are then post‐processed into probability maps which can be used downstream for various quantifications.

In reviewing the repeatability and reproducibility of the tool Dextrusion, we refer to previously published guidelines for publishing image analysis tools by Schmied et al.^2^ For novel workflows, the minimal recommendations include clearly citing all software components and platforms used, describing the sequence of steps and key parameter settings, and providing both example data and code with specific version information. Best practice also involves sharing all settings, making public example datasets available, and providing a rationale for design choices while being transparent about limitations. To further support usability, it is recommended to provide tutorials or screen recordings and distribute the workflow through easy‐to‐install methods, such as containers. For machine learning–based workflows, additional minimal requirements include access to the trained model(s) and validation data. The recommended best practices extend to sharing training and testing data (with metadata), making code openly available, and using cloud‐hosted or containerised distribution. Where possible, models should be provided in standardised formats, such as those supported by the BioImage Model Zoo.^3^

### 1.1 Publishing, sharing and maintaining a tool for the community

The original manuscript, introducing Dextrusion,^1^ is of high quality and follows a clear, logical progression in defining the challenge of identifying cellular events within the increasingly large and complex datasets being generated. The authors report a tool precision of ∼0.8, while emphasising a significant 10‐fold reduction in total processing time compared to manually annotating events.^1^ They also acknowledge limitations of the tool, including variability when using differently labelled samples or tissue types.

Repeatability is well supported: the code is openly available on a moderately documented, institutionally hosted GitLab repository (https://gitlab.pasteur.fr/gletort/dextrusion) shared under a BSD‐3 license, accompanied by additional ImageJ macros and Jupyter notebooks. The tool is also distributed as a Python module installable via Pip for easy installation. Example image datasets and data used for training of the published models are deposited in a Zenodo repository,^4^ and the methods to acquire these datasets are clearly described in the manuscript.

Since its publication in 2023, the GitLab repository has had minor updates with commits aimed at improving documentation (e.g. the main README.md), clarifying usage, and fixing bugs in the provided Jupyter notebooks and ImageJ macros.

## 2 RESULTS

### 2.1 Testing repeatability of Dextrusion

In the lead up to the GloBIAS inaugural meeting, five experts from across the globe worked collaboratively on testing Dextrusion: they independently evaluated the tool by installing and running it on the provided sample dataset and compared notes in online meetings. All testers were able to install the program following the provided documentation. There were challenges in connecting Dextrusion to available discrete graphics cards. These were linked to well‐known issues with TensorFlow GPU support on Windows, which depends on precise combinations of CUDA (11.x) and cuDNN (8.x), and is officially no longer supported for newer versions of TensorFlow. Resolving the issue required manual modifications to system environment variables and compatibility adjustments. Table 1 summarises the testing environments and execution time using the provided sample image file (‘001.tif’; a 20 MB, 60 timepoint, single channel 2D stack, 590 × 297 px in xy) using the *Notum_Ext0* model with default parameters. Across the various hardware Dextrusion was tested on, the times observed to process the provided example dataset were mostly consistent (103–300 s) for non‐GPU execution. In comparison when tested on the same hardware with the GPU enabled saw a significant reduction in processing time (103–16 s). An additional GPU test was performed on a different GPU Card (RTX 1000 Ada generation), which saw an improvement compared to CPU only tests (58 s vs. 103–300 s). However, likely due to the reduced available VRAM cache on the card (6 GB vs. 24 GB for RTX 4090), additional cycling of chunked data added some time cost to the runtime with a reduced computing size of 45,000 used to complete the job. The resulting data outputs were visually and quantitatively consistent across testers and hardware. Therefore, it can be concluded that when using the provided sample data, Dextrusion demonstrates repeatable performance.

**TABLE 1:** Various execution times of Dextrusion on provided sample dataset 001.tif using default parameters using the Notum_Ext0 model.

Test case	Hardware	GPU utilised	Execution time (s)
1	*Virtualised* AMD EPYC 7713 (8 cores, 2.0 GHz), 256 GB RAM, NVidia A40‐48Q	No	162
2	Intel Xeon E‐2276 M (6 cores, 2.8 GHz), 32 GB RAM, NVidia Quadro T2000	No	252
3	Intel Core i5‐8300H (4 cores, 4.0 GHz), 16 GB RAM	No	300
4	Intel Core i7‐12700 (8 cores, 1.6/2.1 GHz), 64 GB RAM, NVidia GeForce RTX 3070	No	120
5	AMD Ryzen 9 7950X (16 cores, 4.5 GHz), 128 GB RAM, NVidia GeForce RTX 4090	No	103
6	AMD Ryzen 9 7950X (16 cores, 4.5 GHz), 128 GB RAM, NVidia GeForce RTX 4090	Yes	16
7	Intel Core Ultra 7 (16 cores, 3.8 GHz), 32 GB RAM, NVidia GeForce RTX 1000 Ada Generation*	Yes	58

*Reduced computing size of 45,000 was required to fit within the GPU memory available.

### 2.2 Testing reproducibility of the model on semi‐synthetic data

In order to test the reproducibility of the Dextrusion tool, we identified a use‐case for the tool with a real‐world dataset acquired within one of our core facilities. A developmental biology group, headed by Melanie White at the Institute for Molecular Bioscience at The University of Queensland, study quail morphogenesis and generously provided access to a dataset for testing purposes. Like many situations when applying code or programs to scientific data, it is not always identical to what may have originally been envisioned. The dataset provided for this test is described previously in the literature.^5^ In the provided time‐series (Figure 1A), nuclei within the developing neural tube region of the quail are expressing Nuc‐mCherry.^6^ During this process, significant 3D cellular migration is occurring, compared to the more 2D development in the drosophila data used in the original Dextrusion paper^1^ and as such significant pre‐processing was required, including image stabilisation using the Correct 3D Drift plugin in Fiji,^7^ and a 2 px median filter was also applied to smooth the data. The dataset available was lacking a membrane channel which is what Dextrusion utilises for cellular segmentation, so a method was devised to produce a semi‐synthetic membrane channel using the find maxima tool in Fiji rather than artificially generate it de novo. First three different nuclei detection algorithms were tested, TrackMate's LoG (Laplacian of Gaussian) detection tool,^7^ CellposeSAM with no user inputs, and CellposeSAM with a defined nuclei diameter of 5 px.^8^ The number of detected nuclei for each timepoint was determined and plotted with 4 manual ground truth counts performed at various timepoints (T0, T15, T30, T45) (Figure 1B and C). The number of nuclei was found to change up and down across the time‐series likely due to residual unstabilised X‐Y movement, and nuclei moving axially during the development process. Nevertheless, these changes in overall nuclei counts and therefore cells should provide examples of cellular division or death events detectable by Dextrusion. TrackMate's LoG detection best matched the manual count data and was chosen to identify nuclei across all timepoints for the downstream membrane channel creation. A 1 × 1 px label image of each detected nucleus was output from TrackMate, these single points were detected using the Find Maxima tool and a synthetic *membrane* channel was produced as the segmented output. The binary image was inverted and a 0.8 sigma Gaussian filter was applied to better match expected input data in Dextrusion (Figure 1D). This new data was then run through Dextrusion using the Notum_All model as it was described in the original manuscript as being the most robust model and therefore would be the most likely best candidate for this novel image series. The program successfully executed on the hardware described above in Table 1 test case 7, using the Dextrusion GUI with the following parameters: cell diameter of 5 px, extrusion duration of 3 frames, computing size of 20,000 and all other settings left as default, taking approximately 4 min to complete without error. The output data was merged with both the input data (synthetic membrane channel) and original nuclei channel (Figure 1Ei) with various detection events for both cell death and cell division observed throughout the time‐course (Figure 1Eii). Note that the sop probability was not tested as these structures are not present in quails. Example high probability candidate cell division events were observed in frames preceding a bifurcation of cellular segmentation (Figure 1Eiii, arrow) as well as for cell death or loss of a tracked cell (Figure 1Eiv, arrow). While other cell death probabilities were detected, their intensities within the heatmap were significantly lower and could be removed with additional filtering; however, due to the complexity of the dataset and associated cellular migrations, this has not been quantified in this review with only qualitative observations made. Despite this limitation (and the fact that we did not re‐train the model), this indicates that Dextrusion could at least *conceivably* be used for different applications. Recent updates to the GitLab repository for Dextrusion includes macros that either cycle through detected events stored in the ROI manager letting the user query if they are true positives or false positives, or can be used to combine the output data with the input data for easier exploration. The former macro attempts to aid the user in event detection by displaying a montage of frames either side of the event; however, in our experience, this only showed the input data channel and not the overlayed probability maps, which would greatly increase its usability.

**FIGURE 1:**
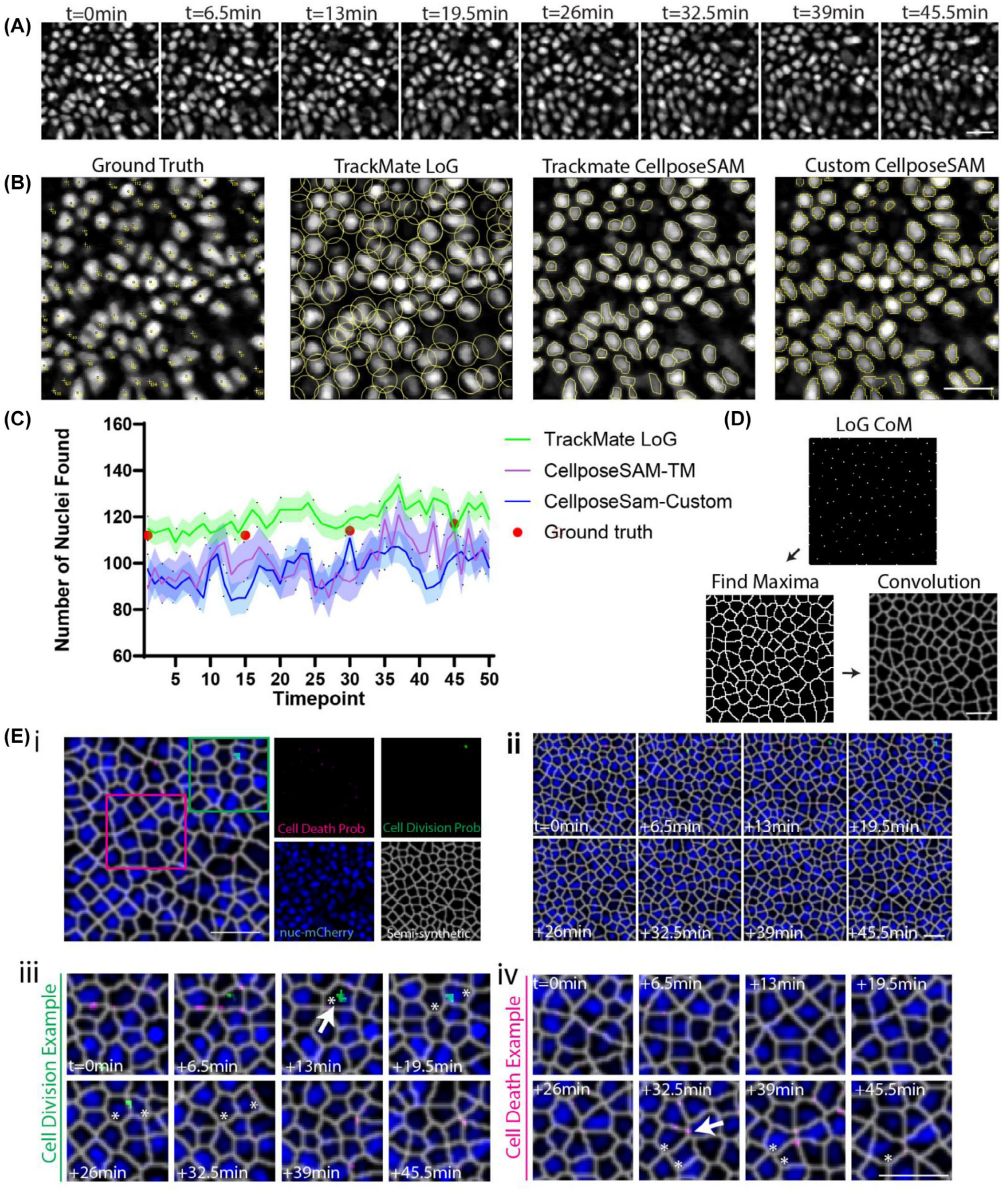
Testing the re‐usability of Dextrusion on novel semi‐synthetic data. (A) Subset of images used to create downstream semi‐synthetic data, time‐lapse confocal acquisition of nuclei labelled with Nuc‐mCherry. Pre‐processing of the data involved image stabilisation using the Correct *3D drift* plugin in Fiji, before a region was cropped and a 2 px median filtered applied. (B) Nuclei detection was evaluated using *TrackMates LoG* detector, cellposeSAM with default settings, and with custom settings (5 px diameter) and compared to manual ground truth counts. (C) Results of time‐series analysis of nuclei count with shaded area representing SD over the entire time‐course for the corresponding method, 4 timepoints were evaluated by manual counting. (D) Semi‐synthetic data processing steps, centre of mass single px points were identified using the LoG detection method, and identified using the *Find Maxima* tool in Fiji. The segmentation output represents a pseudo‐membrane channel for Dextrusion. Following Dextrusion analysis using the *notum_all* model and an estimated cell diameter of 5, (E) probability of cell division (green) and death (magenta) were overlayed with the pre‐processed nuclei (blue) and semi‐synthetic membrane (grey). (i) Sample frame of merged output data, with coloured insets highlighting example event regions below. (ii) Time‐series example showing relative frequency of detected event probabilities. (iii) Example of cell division event (green probability), over a series of frames (interval 6.5 min), showing initial high probability (arrow) and two daughter cells (demarked by *). (iv) Example cell death event (magenta probability), over a series of frames (interval 6.5 min), showing moderate probability (arrow) and a change from two cells to one (demarked by *). Scale bars = 20 µm.

## 3 DISCUSSION

Publishing open‐source software tools for the research community can be a demanding undertaking. Tools must meet high standards of quality, not only to satisfy journal requirements but also to align with community‐driven guidelines for reproducibility, transparency, and usability.^2^ A persistent challenge lies in the long‐term viability of such projects: once the main publication is released, questions of maintenance, sustainability, governance and funding become critical, yet uncertain. Without clear plans for continued support, even well‐designed tools risk becoming difficult to reproduce or extend over time.

### 3.1 Installation of Dextrusion

Reproducing the installation process was largely successful, using the provided instructions, Dextrusion was installed via *pip* on Windows 11 in approximately 10 min, with sufficient guidance available through the GitLab repository. This initial process was straightforward and suggests that the developers have considered accessibility for new users. However, practical challenges emerged when enabling GPU support. After installation, the program did not detect the discrete GPU, and no troubleshooting guidance was available in the documentation. This is a significant limitation, as GPU acceleration is often essential for handling the large datasets typical in this field and as shown above resulted in an almost order of magnitude decrease in execution time on the same dataset and hardware (Table 1).

The steps required to resolve installation challenges related to Tensorflow versions are unlikely to be achievable for non‐expert users without substantial external guidance. These additional hurdles increase the barrier to entry and reduce reproducibility for the broader community, as successful setup becomes dependent not only on the published instructions but also on undocumented technical expertise.

### 3.2 Running Dextrusion

Using the sample data provided alongside the original manuscript all members of the group were able to launch the program, input specific parameters (files paths for local input data and the location of the relevant model file) and rely on the default settings for the remaining options and still achieve an output result. However specific information on how all the various input fields influence the output are lacking in the manuscript or provided documentation.

We did not evaluate the (re‐)training process of the underlying machine‐learning model. The documentation for this seems similar to the wider package's and consists of readme files pointing to Jupyter notebooks and ImageJ macros.

For reproducibility evaluation, we were able to secure data from an independent research lab; however, it lacked the specific membrane channel used in the original manuscript, and was from a different model organism which undergoes significant 3D morphogenesis. To overcome this, we decided to semi‐synthetically develop the membrane channel using tools in Fiji,^7^ this first required nuclei detection, which we tested various available tools and found the LoG detection tool in TrackMate to be the most suited for this dataset. Membrane channel creation was completed using the Find Maxima tool, and was de‐binarised using a Gaussian filter. Using this novel dataset did require some changes to some of the individual settings within the GUI in order for it to run without error (mainly compute size was reduced). While the probability maps identified candidate cell division and cell death events, rigorous event‐level benchmarking would require extensive frame‐by‐frame manual annotation due to the structural complexity and axial movement present in this dataset. Such analysis was therefore considered beyond the scope of the present reproducibility‐focused study. We commend Dextrusion for providing a GUI as it makes the tools accessible to a wider audience. While some comments exist within the GUI for the various input fields, very little information is provided within the documentation, for example, *Computing size* defaults to 150,000, with the comment ‘Computing efficiency, depends on your compute capacity. The higher the faster, but might exceed memory’. Additionally not well explained are the fields *Typical extrusion duration (frames)*, which defaults to 4.5 (whereby frames would normally be a whole integer) or *Min probability volume*, which defaults to 800 (is this in pixels/voxels/microns?). We expect GUI users to require the most guidance to use scientific software appropriately, and therefore highlight this as an area for improvement for Dextrusion (and other scientific software).

Concerningly, within the original published manuscript, the journal Development publishes the *Peer review history* in which, Reviewer #1 states ‘The manuscript is accompanied with open code (looks well organised and documented – I did not try to execute it)’. This highlights another challenge, with editors struggling to find cross‐discipline experts that can critically review and evaluate manuscripts, tools and code, a gap potentially filled by the GloBIAS expert databases or other network organisations.

### 3.3 Evaluation

The manuscript by Villars et al.^1^ when compared to the community checklist,^2^ reasonably meets most of the criteria to the level of best practice. However, some notable omissions mean it cannot receive a perfect score. Challenges with running the tool on novel datasets (due to lack of descriptive documentation) and the trained models not being interoperable with other platforms such as the BioImageZoo^3^ ideally should still be addressed. While the tool can identify candidate cell division and extrusion events, in early testing with the originally published sample data, we were able to achieve similar results with TrackMate^8^ and Cellpose.^9^ This isn't to disparage Dextrusion; it highlights its ability to achieve similar results using alternative methods further validating its utility. Many publications have cited the original manuscript; however, excluding the original authors, none are yet reported *using* the tool, instead of only citing its existence.

## 4 RECOMMENDATIONS/CONCLUSIONS

We can broadly distinguish two purposes for which one might want to share scientific code.

Firstly, to aid reproducibility, it should be possible to reproduce scientific findings. In this case, the provided code is not necessarily intended or guaranteed to be usable beyond reproducing the publication's results, allowing their verification. We commend Dextrusion for the quality and openness of their code, and for fulfilling the majority of the checklist, which we believe clearly exceeds the average publication in the field.

The second purpose of sharing code is to provide it as a tool for others. This requires the software to be broadly usable and maintained over time, necessitating investment in terms of time, funding, and open‐source community engagement. Minimal maintenance seems to have happened since publication of the related article. Long‐term maintainability could be improved by restricting the supported dependency versions, generating API documentation automatically via standardised docstrings, adding unit tests and a contributing guide and using a code formatter (e.g. the ‘ruff’ tool). Additionally, incorporating better descriptions around each user‐adjustable element within the GUI would improve usability of the tool; for example, in an comparative open‐source tool, Cellpose,^9^ text boxes include easy to understand descriptors. For example for the user‐editable variable *cellprob threshold* includes the descriptor ‘threshold on cellprob output to seed cell masks (set lower to include more pixels or higher to include fewer, e.g. in range from (–6, 6))…’ describing its function, the range of values and what the adjustments are likely to achieve.

Overall, we were impressed by the reproducibility and accessibility of Dextrusion. The fact that it has been of further use to the lab where Dextrusion originated is cause for further optimism.^10^ We see risks around its outdated dependency TensorFlow and the lack of a ticketing system or any contribution guidelines for external developers – these limit possible community buy‐in and suggest the software's longevity is tied to its laboratory's need for it. This is typical of research software in general, and we expect this is a consequence of prevalent incentives in academia that don't reward software maintenance or open‐source collaboration.

Dextrusion's shortcomings reflect structural incentives that determine what researchers spend their time on generally. We believe it is important to improve and diversify such career incentives and training for research‐software‐related activities in bioimage analysis (and beyond) to accelerate scientific progress and retain a wider pool of talent in academia. Incentivising cross‐lab collaboration (including transparent governance around giving credit for contributions) on shared research software frameworks (rather than each lab developing their own, possibly incompatible and duplicate piece of software) would further be beneficial for research. This could be done by promoting non‐traditional technical career paths in academia such as bioimage analysts^11^, ^12^, ^13^ and research software engineers^14^, ^15^, ^16^ (possibly based in an imaging facility^17^) might reduce the overall software maintenance burden still too often performed as an afterthought by researchers.

## Data Availability

Data generated as part of this manuscript and review can be downloaded from here: https://doi.org/10.5281/zenodo.18823565
